# Cellular and Molecular Mechanisms Involved in Hematopoietic Stem Cell Aging as a Clinical Prospect

**DOI:** 10.1155/2022/2713483

**Published:** 2022-04-01

**Authors:** Soheila Montazersaheb, Ali Ehsani, Ezzatollah Fathi, Raheleh Farahzadi

**Affiliations:** ^1^Molecular Medicine Research Center, Tabriz University of Medical Sciences, Tabriz, Iran; ^2^Student Research Committee, Tabriz University of Medical Sciences, Tabriz, Iran; ^3^Department of Clinical Sciences, Faculty of Veterinary Medicine, University of Tabriz, Tabriz, Iran; ^4^Hematology and Oncology Research Center, Tabriz University of Medical Sciences, Tabriz, Iran

## Abstract

There is a hot topic in stem cell research to investigate the process of hematopoietic stem cell (HSC) aging characterized by decreased self-renewal ability, myeloid-biased differentiation, impaired homing, and other abnormalities related to hematopoietic repair function. It is of crucial importance that HSCs preserve self-renewal and differentiation ability to maintain hematopoiesis under homeostatic states over time. Although HSC numbers increase with age in both mice and humans, this cannot compensate for functional defects of aged HSCs. The underlying mechanisms regarding HSC aging have been studied from various perspectives, but the exact molecular events remain unclear. Several cell-intrinsic and cell-extrinsic factors contribute to HSC aging including DNA damage responses, reactive oxygen species (ROS), altered epigenetic profiling, polarity, metabolic alterations, impaired autophagy, Janus kinase/signal transducer and activator of transcription (JAK/STAT) pathway, nuclear factor- (NF-) *κ*B pathway, mTOR pathway, transforming growth factor-beta (TGF-*β*) pathway, and wingless-related integration site (Wnt) pathway. To determine how deficient HSCs develop during aging, we provide an overview of different hallmarks, age-related signaling pathways, and epigenetic modifications in young and aged HSCs. Knowing how such changes occur and progress will help researchers to develop medications and promote the quality of life for the elderly and possibly alleviate age-associated hematopoietic disorders. The present review is aimed at discussing the latest advancements of HSC aging and the role of HSC-intrinsic factors and related events of a bone marrow niche during HSC aging.

## 1. Introduction

**Figure 1:**
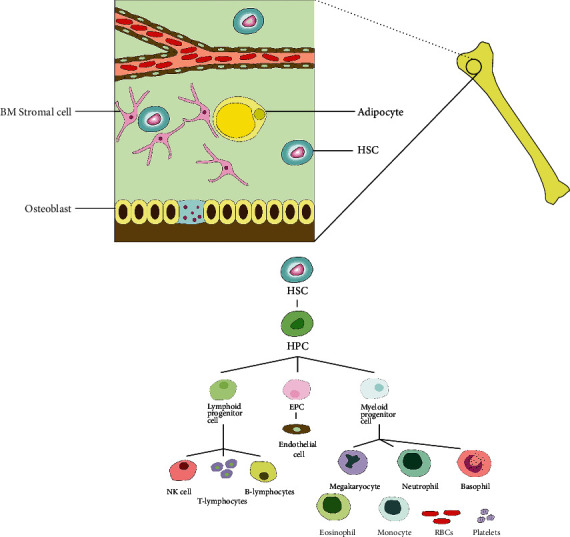
The BM niches. The BM hosts two kinds of adult stem cells, including MSCs and HSCs. The HSCs can give rise to the HPCs which in turn give rise to the lymphoid progenitor cells and the myeloid progenitor cells.

Hematopoiesis is defined as a continuous process by which hematopoietic stem cells (HSCs) replenish diverse types of blood cells such as erythrocytes, B and T lymphocytes, myeloid cells, natural killer (NK) cells, dendritic cells (DCs), mast cells, and platelets during the lifespan of an organism [1, 2] (Figure 1). HSCs are the first isolated and identified stem cells and, more importantly, are still the most studied stem cells. Based on repopulation capacity, HSC pool is divided into three distinct types, including long-term repopulating HSCs (LT-HSCs), short-term repopulating HSCs (ST-HSCs), and multipotent progenitors (MPPs). These progenitors are identified based on cell surface markers and fluorescence-activated cell sorting (FACS) analysis. All murine HSCs are characterized by the lack of lineage-specific surface markers (Lin*^−^*), overexpression of stem cell antigen-1 (Sca-1)^+^, and c-Kit^+^ (LSKs), referring to Lin^−^ Sca1 ^++^ Kit ^+^ or LSK. In addition, it was found that murine HSCs have some primitive markers, including CD48 (Slamf2), CD150 (Slamf1), Flt3, and CD34 [3].

HSCs have the capacity to self-renew and differentiate into diverse types of immune cells, but, similar to adult stem cells, they are susceptible to aging-related stresses. Despite the increasing numbers of human HSCs during aging, a decrease in the self-renewal ability and reconstitution potential of HSCs was observed after transplantation [4]. Upon aging, this gradual loss of the self-renewal and reconstitution potential makes HSCs distinct from pluripotent embryonic stem cells (ESCs) and induced pluripotent stem cells (iPSCs) [5]. Besides, aged human HSCs displayed profound epigenetic reprogramming by targeting cancer pathways, predisposing them to leukemia [6].

Several cell-intrinsic and cell-extrinsic factors contribute to HSC aging. The functional alterations of HSCs with aging are regulated mainly by various cell-intrinsic signals such as DNA damage, reactive oxygen species (ROS), epigenetic changes, and changes in polarity. Furthermore, hematopoietic niche-derived cell-extrinsic factors have a substantial role in the function and maintenance of HSCs [7, 8]. A better understanding of the molecular mechanisms responsible for HSC aging will enable the scientific community to enhance the regenerative capacity and function of healthy HSCs and delay the aging process of the hematopoietic system in the elderly [9].

Given that HSC aging is accompanied by its dysfunction, several studies have investigated the mechanisms behind this. HSC aging is associated with altered expression of some genes and mutations of specific genes [5, 10]. Furthermore, inhibition of specific pathways, such as the mammalian target of rapamycin (mTOR) and p38 mitogen-activated protein kinase (P38 MAPK) signaling pathways, is involved in the aging of HSCs [11]. Additionally, disturbances in epigenetic profiles contribute to the functional decline of HSCs during aging [12]. Various factors within the HSC niche play a crucial role during aging, for instance, cytokines and enzymes [13]. This review compares the distinct biological hallmarks, signaling pathways, and epigenetic profiles of young and aged HSCs. Due to the strong association between hematological malignancies and aging, this review also highlights the relationship between molecular mechanisms and functional alteration and finally may offer important clinical insights.

## 2. Hallmarks of HSC Aging

### 2.1. Repopulation Capacity Defects

It is known that the number of HSCs in bone marrow (BM) is increased by 2 to 10 times as mice and humans age. Nevertheless, the reasons that underlie this aging-associated increase in the HSC number are still vague. This can be due to a possible compensatory mechanism of HSCs to deal with the functional loss [14]. Even though both young and aged HSCs have a similar cell division frequency, an increase in the frequency of symmetric cell divisions may also contribute to an increased number and functional defects in aged HSCs. Besides, several studies indicated that aged HSCs exhibit less quiescence and undergo more cell division [4]; thus, they accumulate more oxidative DNA damage than young HSCs [15]. These factors limit the self-renewal and reconstitution ability of aged human HSCs in the hematopoietic system.

A growing body of evidence showed myeloid-biased differentiation in aged human HSCs [3]. Adelman et al. found that transplantation of young HSCs into aged niches led to homing deficit and reduced differentiation with a bias toward the myeloid lineage [6]. In contrast, there was a limited and incomplete rejuvenation of aged HSCs in the young BM niche [7, 8].

To verify the functional difference between aged HSCs and young, the long-term self-renewal and multilineage capacity of HSCs were determined by a competitive transplantation analysis as a gold standard. In this method, HSCs with BM cells were mixed in order to restore immunity of postirradiation recipient animals [14]. As reported by several investigations, aged HSCs had a diminished repopulation capacity [4]. This evidence implies that the increased number of aged HSCs cannot compensate for immune cells' impaired function and immune homeostasis in aged populations.

### 2.2. Aged HSC Rejuvenation Strategies

HSCs' function declines during aging, but whether this dysfunctionality can be reversible remains vague. Villeda et al. found that exposing old animals with young blood improved the age-related phenotype and reversed preexisting effects of brain aging [16]. This part summarizes some rejuvenation approaches to restore at least the partial function of aged HSC (Table 1). HSC aging is linked with alterations in various gene expressions. The special AT-rich sequence binding protein 1 (Satb1) is an oncogenic driver with potential therapeutic targeting. The reduced level of Satb1 was observed in aged HSCs, and thereby, forced Satb1 overexpression could partially restore the function [17]. In addition, it was found that sirtuins 3 [18] and 7 [19] were suppressed with age. Therefore, upregulation of these regulators might improve the HSC regenerative capacity.

Another approach for rejuvenating aged HSCs relies on the inhibition of the mTOR pathway [20]. mTOR is a crucial regulator of cellular metabolism that acts as a nutrient-sensing and links to cell growth, proliferation, and survival. Nutrient-sensing pathways are a significant determinant of longevity [21]. As mentioned earlier, stem cells are maintained in a quiescent state before activation; thereby, they reduce transcriptional, translational, and metabolic activity by suppressing mTOR activity [22]. Considering the central role of mTOR in age-related disease, inhibition of mTOR by rapamycin or other gene modulatory agents can ameliorate age-related pathologies [23]. It is well known that fasting and refeeding regimens have rejuvenating effects on the hematopoietic system. Cheng et al. reported that extended fasting could regenerate HSCs by reducing protein kinase A (PKA) activity and circulating IGF-1 levels [24]. Moreover, the rejuvenation of aged HSCs can also be affected by diverse pharmacological agents as well as changes in the BM niche, as shown in Table 1.

### 2.3. Homing Defect and Increased Mobilization

Throughout adulthood, HSCs are located in the marrow cavity of all long bones and coexist with other cells in a well-organized structure called niche. It has been revealed that engraftment of HSC into nonmyeloablative recipients led to a spatially localized niche of stem cells. In contrast, other transplanted BM cells became flattened on the bone lining in the periosteum of the bone. Nilsson et al. showed that whole BM transplant containing cells of the bone lineage could engraft and turn into the competent osteoblasts producing the bone matrix [34]. Several lines of evidence demonstrated that osteoblastic cells have a regulatory role in the niche and function of HSCs via the Notch activation pathway [35].

Live imaging-based techniques revealed distinct populations of hematopoietic cells in different regions, depending on their differentiation stage [36]. It is worth noting that transplanted HSCs were more prone to settle in the endosteum of irradiated recipients, while nonirradiated mice had random distributions [37]. Successful treatment of a broad spectrum of blood disorders and malignant diseases such as leukemia, lymphoma, and myeloma relies on the homing and trafficking ability of donor HSCs into the BM of the host [38]. Liang et al. reported harmful effects of aging on homing ability and engraftment of HSCs. According to their findings, aged mouse HSCs had a threefold lower homing efficiency than young HSCs [39].

Another similar report displayed the decreased homing potential of aged HSCs in BM compared to the young counterparts [40]. Systemically administered cytokines or cytotoxic agents could induce mobilization of HSCs from the BM into the peripheral blood (PB), which subsequently could be collected for HSC transplantation and treatment of immune deficiencies and malignancies [41]. A body of growing evidence has revealed the crucial role of Granulocyte Colony-Stimulating Factor (G-CSF) in mobilizing hematopoietic cells from the BM into the PB. It was reported that mice treated with G-CSF exhibited a higher level of all lineage progenitors in the spleen [42, 43]. In other words, hematopoietic progenitor cell (HPC) mobilization was noticeably impaired in mice deficient with the G-CSF receptor (G-CSFR). Given the expression of G-CSFR on mature hematopoietic cells, it can be assumed that G-CSFR signals have a fundamental role in HPC mobilization [42]. However, Liu et al. reported that G-CSFR expression on HPCs was not necessary for their mobilization, indicating the indirect effect of G-CSF on hematopoietic cells for HSC mobilization [44].

Xing et al. reported that upon stimulation with G-CSF, mobilization of hematopoietic stem and progenitor cells (HSPCs) from BM into the PB was strongly dependent on deadhesion of HSPCs from the niche. They showed that aged mice exhibited a 5-fold increase in HSC mobilization in a mouse model of G-CSF-induced mobilization [45].

### 2.4. Skewing in Lineage Distribution

Under the normal physiologic conditions, HSCs differentiate into myeloid and lymphoid lineages, maintaining a balanced pattern and controlled production. On the other hand, a higher prevalence of anemia and compromised adaptive immunity occur in older adults. The reasons behind this are related to the impaired function of T and B lymphocytes due to the involution of the thymus and a low number of aged lymphoid progenitors [46]. Indeed, aging can drive HSC differentiation toward myeloid lineage with high myeloid cells in PB. There is a severe upregulation in the age-associated genes in myeloid malignancies [47]. During aging, myeloid cells are preserved, while B lymphoid cells are decreased, resulting in a skew in the myeloid to lymphoid ratio (myeloid/lymphoid) [48]. This skewing may explain a higher incidence of myeloid versus lymphoid malignancies in aged subjects [49].

In this context, Sudo et al. reported that despite less differentiation in aged HSCs, they still exhibited self-renewal potential to regenerate blood cells. According to their study, HSC levels gradually increased with age due to the constant self-renewal of HSCs [50]. As evidenced by aged mice, myeloid progenitor numbers showed relative expansion compared to the young mice, a characteristic of aged HSCs known to be cell autonomous [51]. Collectively, the expression of myeloid-specific genes is upregulated during HSC aging, whereas lymphoid-specific genes are downregulated [52].

### 2.5. The Cell-Intrinsic Mechanisms of HSC Aging

**Figure 2:**
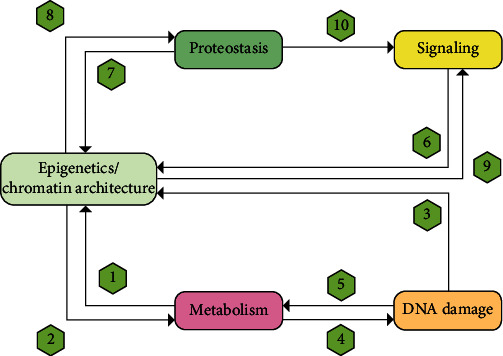
Interconnections between different biological processes involved in intrinsic HSC aging.

As discussed earlier, the decline of HSC functioning with age is thought to be driven by a variety of molecular and cell-intrinsic mechanisms [14]. Although mechanistically, it is possible to separately discuss these multiple aging pathways, they are highly interconnected and interdependent (Figure 2).

### 2.6. DNA Damage Responses and Genetic Mutations in HSC Aging

Unlike proliferating progenitors, which rely on reliable homologous recombination (HR) pathways to repair DNA damage, quiescent HSCs use the error-prone nonhomologous end joining (NHEJ) repair pathway, making them prone to DNA damage [53]. Several studies have demonstrated an increase of 2-3-fold in accumulated DNA damage in aged HSCs, as identified by staining of H2A histone family member X (H2AX) foci, DNA mutation frequency, the alkaline comet assay, and the LOH assay [54–56]. Relying on these findings, it can be explained that the elderly are more likely to acquire mutations, age-related clonal hematopoiesis, and a higher risk of myeloid malignancies [57, 58]. It was identified that DNA damage has a crucial role in driving HSC aging. It was evidenced by the premature aging phenotype of HSCs isolated from mice lacking the DNA repair components [59, 60].

DNA damage in HSCs may result from errors during DNA synthesis or/and by endogenous factors, such as elevated ROS levels or environmental stressors [61]. Indeed, DNA damage impairs HSC function by inducing DNA damage repair and activating cell cycle checkpoints such as CD53-p21-mediated cell cycle arrest [62]. Besides, overexpression of senescence-associated of p16Ink protein [15] and proapoptotic proteins such as PUMA (as an essential factor for p53-dependent apoptosis) [53] can impair HSC function. Beerman and his colleagues showed that HSCs isolated from old mice had consistent evidence of DNA strand breaks, demonstrating that HSCs are not uniquely genoprotected with age [56]. According to a recent report, aged HSCs also displayed more replication errors [63].

### 2.7. Reactive Oxygen Species

In the BM, HSCs are located within hypoxic niches which may protect them against oxidative stress and promote self-renewal potential [64]. Since HSCs are quiescent and maintain low metabolic requirements, they produce low levels of ROS. However, it has been shown that ROS levels increase as HSCs age, resulting in oxidative stress in HSCs [65, 66]. In addition to ROS levels increasing during aging, it also contributes to increased proliferation rate, senescence, and apoptosis. The self-renewal potential of HSCs exposed to low ROS levels was higher, as evidenced by serial transplantations. By contrast, exposing HSCs to a higher level of ROS results in self-renewal failure, accompanied by upregulation of mTOR and p38 mitogen-activated protein kinase activity [67].

According to a study in the three mouse models (young, middle, and aged), mitochondria and NADPH oxidase were the main ROS-generating sources in the three groups, while cytochrome P450 contributed to the aged and middle groups and xanthine oxidase only to the aged one. Besides, DNA damage and apoptosis were detected in the middle and aged mice. Also, old mice exhibited shorter telomere length. As evidenced, telomere shortening occurs with age, playing an essential role in myeloid skewing [68]. With these backgrounds, oxidative stress might contribute to HSC dysfunction during the aging process [69, 70].

Previous publications have reported that ROS plays a significant role in regulating HSC aging. It has been found that transcription factors of forkhead box O (FOXO) family such Foxo1, Foxo3a, and Foxo4 have an essential role in regulating HSC pools, progenitors, and ROS-mediated activity in HSCs [71].

Several lines of evidence revealed overexpression of hypoxia-inducible factor-1*α* (HIF-1*α*) in HSCs. Interestingly, HIF-1*α* could switch HSC cellular metabolism from mitochondrial respiration into glycolysis, ultimately reducing ROS production. Indeed, HIF-1*α* deletion in HSCs could induce ROS generation and negatively impact long-term repopulation ability [72].

### 2.8. Altered Epigenetic Profiling

The term epigenetics refers to changes in gene expression without affecting the DNA sequence. In other words, it is a change in phenotype without changing the genotype. Epigenetic regulation is a key mechanism that maintains the multipotency and self-renewal of HSCs. This process is mediated by DNA methylation or histone modification (methylation/acetylation) to preserve self-renewal gene expression and suppress involved genes in differentiation and lineage fate [73, 74]. It is well documented that DNA methyltransferase 1 (Dnmt1) is a crucial regulator of HSCs and exerts its effect by reestablishing existing DNA methylation profiles during the cell replication. This is possibly mediated by recognizing hemimethylated DNA and maintaining preexisting DNA methylation patterns of the parent strand on the daughter strand [75]. Dnmt3a/3b is involved in de novo DNA methyltransferase and establishes new DNA methylation during the development and differentiation of stem cells [76, 77].

Compelling evidence indicates that Dnmt1-deficient mice had a reduction in HSC number and function [78]. However, in Dnmt3a-knockout mice, HSCs could grow and self-renew more efficiently and are surprisingly enhanced in mice with Dnmt3a/3b double-knockout [76, 79]. A growing body of study suggests that altered epigenetic profiles are strongly associated with HSC aging. The global DNA methylation of old HSCs is generally stable or slightly higher than that of young HSCs. These epigenetic alterations could affect not only self-renewal genes but also contribute to age-dependent functional decline and myeloid-biased differentiation. This is possibly due to the regulation of gene expression levels in differentiated progeny [6, 80]. In addition, age-related epigenetic alterations of HSCs are strongly linked with a proliferation history, indicating that epigenetic memory loss is driven by proliferation [12]. A proliferation-driven HSC aging occurs by switching HSCs from a dormant state and multipotency to activation and lineage priming. This process is mediated by a series of factors through inducing the epigenetic switch such as Ezh1-to-Ezh2 PRC2 [80], decreasing the level of Dnmt1, Dnmt3b, and all three Tet enzymes, as well as the involvement of critical modulators of chromatin states such as Bmi, Eed, Suz12, Jarid1b, Kat6b, Sirt1, and Suv39H1 [80, 81].

As discussed earlier, aged human HSCs have a profound epigenetic reprogramming by targeting cancer-related pathways, predisposing to leukemia [6]. In this context, it was reported that redistribution of DNA methylation and decrease in H3K27ac, H3K4me1, and H3K4me3 levels predisposed cells to age-related acute myeloid leukemia (AML) as compared to the young HSCs [82].

### 2.9. Polarity

Asymmetric distribution of specific proteins known as “increased polarity” has been recognized as a prominent characteristic of aged HSCs, while this feature is less obvious in young HSCs [83]. The cell division control protein 42 homolog (Cdc42) is believed to be responsible for the unequal distribution of these proteins. Cell Cdc42 switches an inactive GDP-bound state to an active GTP-bound state in response to different signals. Besides, this molecule can regulate actin polymerization and organization of tubulin, affecting cell-cell and cell-extracellular matrix adhesion and the polarity in various cell types [27, 83]. With this notion, the application of Cdc42 inhibitors can restore the polarity in aged HSCs and improve their function after transplantation [84]. According to Florian et al., the elevated activity of Cdc42 is linked to the aging of HSCs and the loss of polarity of aged HSCs [85]. In aged HSCs, constitutive activation of Cdc42 induced premature aging of HSCs by depolarizing Cdc42 and tubulin. Pharmacological inhibition of Cdc42 activity could restore the cellular function of aged HSCs [86], although it is not clear whether the acute reversal of asymmetry in protein distribution has long-term effects on the function of HSC.

### 2.10. Metabolic Alterations and Impaired Autophagy

**Figure 3:**
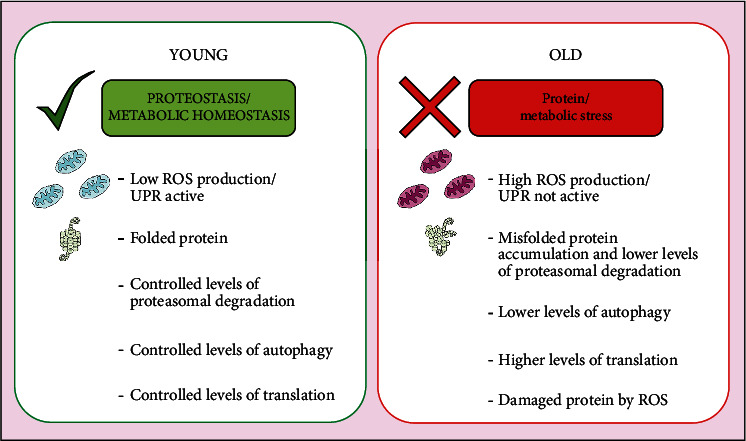
Metabolic homeostasis and proteostasis during aging in HSC.

As described above, HSCs maintain a low metabolic rate and glycolytic activity. A young HSC undergoes an oxidative metabolic change following activation, which can be reversed by returning to the quiescent state. In contrast, the basal metabolism of aged HSC shifts towards oxidative metabolism [87], which leads to an increase in ROS levels and a decrease in regenerative capacity [88]. As a response to metabolic stress, cells engage autophagy, a “housekeeping” mechanism for the self-degradation of cellular components [89]. In this process, organelles or portions of the cytosol are enclosed within double-membrane vesicles, which are subsequently fused with the lysosome where degradation occurs [90]. It has been well documented that the deregulation of autophagy is associated with aging and various age-related diseases such as cancer [91] (Figure 3).

## 3. Alterations in the Intrinsic Signaling Pathways Present in HSC Aging

**Figure 4:**
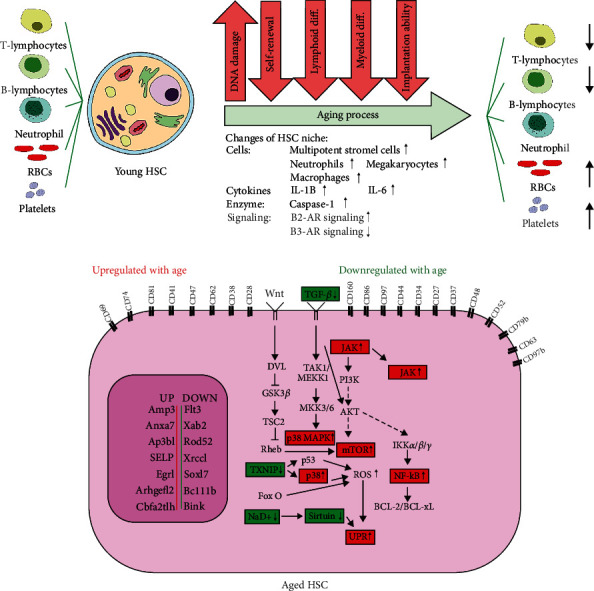
Functional alterations and HSC aging mechanisms. Aging affects HSC functions, including decreasing self-renewal ability and myeloid/platelet-biased differentiation and impairing implantation ability. The intrinsic mechanisms are illustrated at the gene level, signaling pathway level, and epigenetic level.

Several studies have found that age-related decline in the functional capacity of HSCs is associated with multiple signaling pathways. Signaling pathways that contribute to HSC aging include Janus kinase/signal transducer and activator of transcription (JAK/STAT) pathway, nuclear factor- (NF-) *κ*B, mTOR, transforming growth factor-beta (TGF-*β*), and wingless-related integration site (Wnt) (Figure 4) [92].

### 3.1. The Signaling Pathways of JAK/STAT, NF-*κ*B, and mTOR involved in HSC Aging

JAK/STAT signaling cascade is a highly conserved event that regulates biological processes such as immune responses, regeneration, and homeostasis [93]. Besides, this pathway controls stem cell dynamics and senescence. Using a single-cell transcriptome, a previous study by Kirschner et al. found that the JAK/STAT signaling pathway had a crucial role in stem cell exhaustion during aging. They detected a divergent subpopulation of old HSCs with a p53 signature. p53 has a substantial role in hematopoietic aging. Increasing p53 activity decreases the function and proliferation of HSCs, while decreasing p53 levels has the opposite effect. The relationship between p53 signaling and JAK/STAT was investigated through constitutive activation of JAK2 (V617F) and p53-positive expansion in aged mice. JAK2- (V617F-) mediated proliferative activity showed a critical role in the p53-induced functional decline in aged HSCs [94]. Additionally, it is well established that NF-*κ*B-mediated activity has a substantial regulatory role in HSC aging [95]. In a study carried out by Stein et al., they identified the role of the NF-*κ*B subunit RelA/p65 in HSC regulation in mice lacking RelA/p65. p65 is the main regulator of hematopoietic development [96]. Loss of p65 led to a severe functional defect in HSCs. Besides, there was an increase in HSPC cycling, differentiation deficits, and extramedullary hematopoiesis [97]. Chen and Kerr reported that aged HSPCs exhibited elevated activity of NF-*κ*B that resulted in increased differentiation and loss of self-renewal [74]. Rad21/cohesin is a critical mediator of NF-*κ*B signaling and is necessary for normal differentiation; however, it can limit HSCs' self-renewal during the aging process in an NF-*κ*B-dependent manner. In this context, old HSCs displayed failure in downregulating Rad21/cohesin and differentiation signals. Collectively, these findings indicate that aged HSCs have increased NF-*κ*B activity [98].

As discussed in the previous part, the mTOR pathway is a robust regulator of cellular function that integrates a wide variety of signals received from mitogens, nutrients, and energy levels [99]. It is well accepted that mTOR inhibition enhances lifespan, but the mechanism of action by which this occurs is still vague. Growing evidence has shown that hyperactivity of mTOR is strongly linked with age-associated disorders [20]. Furthermore, several studies have demonstrated that mTOR inhibition with rapamycin attenuated the pathological processes [100]. Bitto et al. reported that three months of rapamycin therapy could inhibit the mTOR pathway and extend the lifespan up to 60% in middle-aged mice [101]. Chen et al. found that HSCs from aged mice had higher phosphorylated (p-)mTOR and mTOR activity than those HSCs from young mice. According to their results, intraperitoneal injection of rapamycin at a dose of 4 mg/kg every other day for six weeks in aged mice extended the life span. These data implicated the efficacy of rapamycin in restoring HSC functions in the old mice [102].

### 3.2. The Role of the TGF-*β* Signaling Pathway in HSC Aging

The transforming growth factor-*β* (TGF-*β*) pathway is fundamental for many cellular functions. This pathway also regulates HSC features such as self-renewal, differentiation, and quiescence [103]. Given the regulatory role of TGF-*β* potential in differentiation among HSC subtypes, Challen and colleagues reported that TGF-*β*1 could stimulate the proliferation of myeloid-biased HSCs in young mice and prevented the turnover of lymphoid-biased HSCs [104].

On the contrary, it was demonstrated that old mice treated with TGF-*β*1 produced fewer myeloid cells. Indeed, aged HSCs exhibited more responsiveness to TGF-*β*1 than young HSCs [105]. Genome-wide transcriptome analysis during HSC aging demonstrated the downregulation of regulatory genes involved in the TGF-*β* pathway (Smad4, Nr4a1, Endoglin, Cepba, Jun, Spectrin b2, and Junb), indicating a decline of TGF-*β* signaling with aging [106].

### 3.3. The Wnt Pathway

As described earlier, polarity is linked with specific functions of HSC, including migration and division. However, polarity loss is associated with an impairment of self-renewal capacity, accompanied by alteration in HSC differentiation [92]. In addition, there is an elevated level of Cdc42 in aged HSCs, which is associated with loss of polarity [86]. According to further research on the Cdc42 polarity pathway, HSC aging is caused by changes in Wnt signaling, from canonical to noncanonical [107]. It has been identified that treatment with Wnt5a led to a series of events including activation of Cdc42, induction of aging-associated polarity, a decline in regenerative potential, and modification of myeloid-lymphoid differentiation in young HSCs [107].

### 3.4. Other Pathways

G-CSF transiently upregulates stromal cell-derived factor-1 (SDF-1) and activates CXC chemokine receptor-4 (CXCR4) signaling that results in hepatocyte growth factor (HGF) production. HGF can bind to c-Met and activate c-Met signaling, regulating the mTOR-FOXO3a signaling pathway [108]. Furthermore, G-CSF signaling can facilitate ROS production and HSC egress from BM [109].

## 4. HSC Aging Occurs through Changes in the Extrinsic Factors

In addition to intrinsic mechanisms, some studies have found that extrinsic factors also contributed to HSC aging [110]. HSC function is strongly affected by the BM microenvironment. Megakaryocytes promote HSC quiescence within this niche [111]. Li et al. provided insights regarding the mechanism of homing HSPC. They reported that the vascular cell adhesion molecule-1^+^ (VCAM-1) macrophage with patrolling properties interacted with and homed HSPCs into a vascular niche [112]. In another study by Chow et al., CD169^+^ macrophages in the BM enhanced the retention of HSPCs [113]. Furthermore, Winkler et al. demonstrated that phagocytic macrophages with the unusual F4/80^+^Ly^−^6G^+^CD11b^+^ phenotype could maintain HSC niches, and more importantly, the loss of these macrophages could mobilize HSCs [114]. A previous study found that regulatory T cells that highly expressed CD150 could maintain a quiescent state, HSC numbers, and immune privilege by the adenosine pathway [115]. Along with the hematopoietic cells mentioned above, several nonhematopoietic cells such as mesenchymal stromal cells (MSCs), perivascular cells, and arterial and sinusoidal endothelial cells have a pivotal role in the HSC niche [116, 117].

The sympathetic nervous system (SNS) regulates HSC trafficking and orchestrates adrenergic neurotransmission into the microenvironment on circadian rhythms [118]. Here, we summarize how HSC environment, the SNS, and other related factors affect HSC aging.

The research of Maryanovich et al. in 2018 proved that HSC aging significantly relied on the innervation of the BM by SNS, since the loss of SNS nerves or adrenoreceptor *β*3 signaling (ADR*β*3) resulted in premature HSC aging. Remarkably, in an in vivo setting, supplementation of a sympathomimetic with selective effect on ADR*β*3 significantly rejuvenated the function of aged HSCs. These findings suggested that maintenance of SNS innervation of BM may offer new strategies for HSC rejuvenation [119].

Megakaryocytes also exhibit the potential of inhibiting HSC proliferation. As mentioned above, both HSCs and megakaryocytes increase during aging. HSCs are located further from megakaryocytes; thereby, it seems that decreased interactions between HSCs and megakaryocytes may be involved in premature hematopoietic aging. In other words, the distance between HSCs and megakaryocytes could regulate HSC proliferation and enhance ADR*β*3 during physiological aging. In this regard, Ho et al. identified that *β*-adrenergic signals promoted megakaryopoiesis during aging. They showed that HSC-supporting niches declined near the bone during natural aging; however, they expanded away from it. Increasing noradrenergic innervation of the BM raises interleukin-6-dependent megakaryopoiesis through the *β*2-adrenergic receptors (ARs). Besides, reduction of *β*3-AR-Nos1 activity is associated with niche alterations in aging, leading to myeloid expansion and impaired lymphoid differentiation [120]. Frisch et al. also found that the dysfunction of aged macrophages was associated with HSC platelet bias and an increase in senescent neutrophils in aged mice compared to younger counterparts. Aged macrophages from the marrow of old mice and humans displayed an activated phenotype and overexpression of inflammatory markers such as IL-1*β*. Altogether, it can be assumed that overexpression of IL-1*β* and caspase-1 in the aged mouse BM niche has a contributory role in age-related lineage skewness of HSCs [13].

## 5. Conclusion

This paper summarizes the hallmarks of HSC aging pertaining to repopulating capacity, homing ability, mobilization, and lineage skewing. Multiple cell-intrinsic factors contribute to HSC aging, such as genetic mutations and DNA damage, ROS production, epigenetic alterations, polarity, clonality, metabolic changes, and impaired autophagic activity. Numerous studies using knockout and transgenic animal models have demonstrated that epigenetic factors are crucial for maintaining proper HSC function. In general, several cell-extrinsic factors, such as HSC-surrounding niches such as megakaryocytes, MSCs, macrophages, and neutrophils, impact HSC aging. *β*-Adrenergic nerve signals, cytokines such as IL-6 and IL-1*β*, and enzymes like caspase-1 also influence HSC aging. Furthermore, inhibition of specific pathways, such as the mTOR and P38 MAPK signaling pathways, is involved in the aging of HSCs.

The interconnections between these processes will be crucial in deciphering how aging affects stem cells. Most of the aging mechanisms reviewed in this paper have been investigated in mouse or nonhuman systems. However, we would like to highlight the progress that has been made to date and the importance of pursuing an integrated approach to connect all underlying factors that affect HSC upon aging. Accordingly, a more comprehensive perspective regarding this process might be the key to bridging the gap between translation and the human system. Therefore, future work should emphasize the mechanisms of the HSC niche during aging. Moreover, expanding long-term HSCs *in vitro* is still a challenge, and the findings of HSC aging could be applied to this challenge.

## Figures and Tables

**Table 1:** Rejuvenation approaches in aged HSCs.

Rejuvenation approach	Mechanism of action	Outcomes	Ref
Satb1 upregulation	Genetic modulation	Promote reconstituting and lymphopoietic potential of aged HSCs	[17]
Sirtuin 3 upregulation	Genetic modulation	Enhancement of the regenerative potential of aged HSCs	[18]
Sirtuin 7 upregulation	Genetic modulation	Restoring mitochondrial dysregulation Reduce myeloid bias	[19]
Curcumin	Pharmacological modalities	Boost the regenerative potential of aged HSCs Restore the engraftment ability	[25]
Microvesicles from young MSC	Pharmacological modalities	Rejuvenate the aged HSCs Restore function via transferring microvesicles containing autophagy-related mRNAs	[26]
Extended fasting	Pharmacological modalities	Decreasing circulating IGF-1 levels and PKA activity	[24]
cdc42 inhibitor (CASIN)	Pharmacological modalities	Promote rejuvenation capacity of the HSC Reverting a normal phenotype Restore the cellular function of aged HSCs	[27]
p38/MAPK inhibitor (TN13)	Pharmacological modalities	Rejuvenating aged HSCs through reducing ROS	[28]
p38/MAPK inhibitor (SB203580)	Pharmacological modalities	Restore the repopulating potential Maintenance of HSC quiescence	[29]
BCL-2and BCL-xL inhibitor (ABT263)	Pharmacological modalities	Depletion of senescent HSCs Improve reconstitution potential	[30]
mTOR inhibitor (rapamycin)	Pharmacological modalities	Increasing regenerative capacity of HSCs Extending the life span	[31]
RANTES/CCL5 knockout	Targeting the BM niche	Decrease myeloid bias Improve the engraftment potential after transplantation	[32]
Bone marrow transplant	Changing the BM niche	Restoring the normal phenotype	[33]

Special AT-rich sequence binding protein 1: Satb1; cell division control protein 42 homolog: Cdc42; mammalian target of rapamycin: mTOR.
